# Effects of NSAIDs on the Release of Calcitonin Gene-Related Peptide and Prostaglandin E_2_ from Rat Trigeminal Ganglia

**DOI:** 10.1155/2017/9547056

**Published:** 2017-10-25

**Authors:** Vittorio Vellani, Giorgia Moschetti, Silvia Franchi, Chiara Giacomoni, Paola Sacerdote, Giada Amodeo

**Affiliations:** ^1^Dipartimento di Scienze Biomediche, Metaboliche e Neuroscienze, Università di Modena e Reggio Emilia, Via Campi 287, 41125 Modena, Italy; ^2^Dipartimento di Scienze Farmacologiche e Biomolecolari, Università degli Studi di Milano, Via Vanvitelli 32, 20129 Milano, Italy; ^3^Dipartimento di Economia, Scienze e Diritto, Salita alla Rocca 44, 47890 San Marino, San Marino

## Abstract

Nonsteroidal anti-inflammatory drugs (NSAIDs) are frequently used to treat migraine, but the mechanisms of their effects in this pathology are not fully elucidated. The trigeminal ganglia and calcitonin gene-related peptide (CGRP) have been implicated in the pathophysiology of migraine. The release of CGRP and prostaglandin E_2_ (PGE_2_) from freshly isolated rat trigeminal ganglia was evaluated after oral administration of nimesulide, etoricoxib, and ketoprofen, NSAIDs with different pharmacological features. Thirty minutes after oral administration, nimesulide, 10 mg/Kg, decreased the GCRP release induced by an inflammatory soup, while the other NSAIDs were ineffective at this point in time. Two hours after oral nimesulide (5 and 10 mg/Kg) and ketoprofen (10 mg/Kg), but not of etoricoxib, a significant decrease in the CGRP release was observed. All drugs reduced PGE_2_, although with some differences in timing and doses, and the action on CGRP does not seem to be related to PGE_2_ inhibition. The reduction of CGRP release from rat trigeminal ganglia after nimesulide and ketoprofen may help to explain the mechanism of action of NSAIDs in migraine. Since at 30 minutes only nimesulide was effective in reducing CGRP release, these results suggest that this NSAID may exert a particularly rapid effect in patients with migraine.

## 1. Introduction

Migraine is a very common and disabling neurological disorder, with a complex pathophysiology that has not yet been fully understood. In current hypotheses, migraine-specific triggers cause a primary brain dysfunction, which causes dilation of cranial blood vessels innervated by trigeminal fibers [1]. The dilated blood vessels mechanically activate perivascular trigeminal sensory fibers to release vasoactive peptides such as substance P and calcitonin gene-related peptides (CGRP). These and other peptides further increase vasodilation and produce neurogenic inflammation, leakage of blood vessels, and mast cell activation [2]. CGRP is a 37 amino acid neuropeptide widely expressed within the nervous system both centrally and peripherally in every site that has been implicated in migraine: meninges, trigeminal ganglia, spinal cord trigeminocervical complex, brainstem nuclei, and cortex [3, 4]. In the trigeminal vascular system (the portions of the trigeminal nerve that innervate cerebral and meningeal blood vessels), more than 50% of trigeminal neurons express CGRP [5]. Upon stimulation, this peptide is released from the neuronal cell bodies in the trigeminal ganglia and at the nerve endings, where it mediates vasodilation via smooth muscle cell receptors and plasma extravasation [6]. Isolated trigeminal ganglia release CGRP both basally, and in response to noxious stimuli and to inflammatory mediators, with a rapid response [5]. CGRP and CGRP receptors are also present centrally, where the peptide may participate in central sensitization [7]. Although there are several other peptides and factors coreleased by trigeminal nociceptor endings, CGRP is recognized as crucial in the pathophysiology of migraine [8]. New compounds that target CGRP or its receptor have been increasingly explored in recent years as new treatments for migraine [4, 8, 9].

About half of migraine patients recur to over-the-counter medications to treat their acute attacks instead of getting professional advice on available specific treatments [10], and nonsteroidal anti-inflammatory drugs (NSAIDs) are still largely used to treat migraine [11]. Although NSAIDs have been used clinically for many years, and it is well known that their effects involve inhibition of the two cyclooxygenases (COX-1/-2) expressed throughout the body, it is not clear which mechanism, apart from the established inhibition of prostaglandin synthesis, defines their different clinical profiles, which are highly relevant for the treatment of migraine in patients. Comparative studies between NSAID effects in headache or migraine models are only few [12, 13]. In particular, very little is known about the effect of NSAIDs on CGRP in the trigeminal vascular system, but some recent evidence indicate that both nonselective and COX-2-selective inhibitors in vitro are able to block CGRP release from cultured TG cells [13]. The objective of our experimental study was to evaluate whether different NSAIDs modulate trigeminal CGRP basal and induced release, an action that would be highly significant in the control of migraine. At the same time, we were interested in testing the possible relation between the inhibition of prostaglandin E_2_ (PGE_2_) synthesis and that of CGRP release in trigeminal ganglia (TG). To this purpose, we tested the effect of *in vivo* administration of nimesulide, ketoprofen, and etoricoxib, 3 NSAIDs with different selectivity for COX-1/-2 on the release of CGRP and PGE_2_ from rat TG, in basal conditions and during stimulation with an artificial inflammatory soup. Freshly isolated TG, where trigeminal sensory neurons are still within their natural environment of supportive cells, are considered the most suitable preparation for the purpose of our study.

## 2. Materials and Methods

### 2.1. Animals

Sprague Dawley male adult rats (weight 250–300 g) were purchased from Envigo, Italy and housed with light/dark cycles for 12 hours, temperature of 22 ± 2°C, humidity of 55 ± 10%, and food and water ad libitum. After at least 1 week of acclimation, the animals were randomly assigned to the different treatments. They were sacrificed following total anesthesia according to European Community Council Directive 86/609/EEC on the ethical use of animals, following protocols according to the guidelines of the Committee for Research and Ethical Issues of the International Association for the Study of Pain. Experimental procedures and research project were approved by local institutional animal care and use committee.

### 2.2. Drugs and Treatments

Nimesulide, a preferential COX-2 inhibitor, was obtained by Helsinn Healthcare SA (Lugano, Switzerland); ketoprofen, a NSAID with a high COX-1 inhibition activity, and etoricoxib, a selective COX-2 inhibitor, were purchased from Sigma (Milan, Italy). Drugs were dissolved in water, sonicated while stirring immediately before administration, and delivered by oral gavage (per os) in a volume of 1 ml/Kg. Control animals were treated with the same volume of vehicle (water) only. Animals were then returned to their individual cage with free access to food and water. Animals were monitored to ensure the absence of any treatment regurgitation, which is however unlikely in the rat. All drugs were administered at the doses of 10 mg/Kg, 5 mg/Kg, and 1 mg/Kg. These doses were chosen on the basis of the previous literature data that showed a significant anti-inflammatory activity in rat models [14–16]. Either 30 minutes or 2 hours after oral NSAID administration, animals were sacrificed with CO_2_ for trigeminal ganglia collection.

### 2.3. Trigeminal Ganglia Isolation and Preparation

Trigeminal ganglia were rapidly dissected with a standardized procedure taking between 5 and 7 minutes. The meninges were removed, and each ganglia was chopped into 4 pieces of 1.5–2 mm thickness in Ca^2+^-free PBS at room temperature, to facilitate diffusion of CGRP and PGE_2_ released from trigeminal cells. Chopped ganglia were then rinsed for 5 minutes with oxygenated Dulbecco's Modified Eagle's medium (DMEM, no serum and no glutamine added) at 37°C in an Eppendorf tube of 1.5 ml volume, which was exchanged 5-6 times. This tissue preparation was chosen after preliminary experiments, as it proved to represent a good compromise to keep as intact as possible the ganglionic architecture and at the same time improve the release of PGE_2_ and CGRP into the extracellular solution. After rinsing, a 0.5 ml volume of DMEM was added to the ganglia and placed in a CO_2_ incubator at 37°C. After 5 minutes, the supernatant was removed after gentle stirring and used for measuring basal release. Medium was immediately replaced with 0.5 ml of an artificial inflammatory soup (IS): 10 *μ*M serotonin, 10 *μ*M histamine, 10 *μ*M bradykinin, 25 mM KCl, and 10 *μ*M capsaicin, dissolved in DMEM. After 5 minutes and a final gentle stirring, supernatant was removed. Basal and IS samples were centrifuged to remove small debris and immediately split in 2 aliquots, one was immediately stored at −80°C for PGE_2_ quantitation, while the other one was diluted with 25 *μ*l of 10x EIA buffer from the CGRP EIA Kit. No reliable CGRP measurement could be performed if samples did not contain EIA buffer before freezing. Also, these samples were then stored at −80°C before analysis.

### 2.4. CGRP and PGE_2_ Assays

CGRP and PGE_2_ were evaluated with commercially available EIA assay kit (SpiBio cat. number A05482 and Cayman cat. number 514010, resp.). Sensitivities of the assays were 1 pg/ml for CGRP and 15 pg/ml for PGE_2_.

### 2.5. Statistical Analysis

Statistical analysis was performed using GraphPad Prism 5 Software (San Diego, CA, USA). Data were tested for equal variance and for normal distribution before choosing statistical tests. Analysis was performed with one way ANOVA. If an overall test comparing groups was significant, Bonferroni test was used for between-group comparisons in the post hoc analysis. The overall significance level was 0.05 for each hypothesis. Each group consisted of 6 animals. Data are mean ± SEM.

## 3. Results

Rats were treated orally with nimesulide, ketoprofen, etoricoxib, or water, and 30 minutes or 2 hours after administration, TG were dissected and isolated as described in Methods. TG were rinsed in Dulbecco's Modified Eagle's medium at 37°C, then were allowed to rest for 5 minutes in a 500 *μ*l volume of fresh medium, which was then collected for evaluation of basal CGRP and PGE_2_ release. Removed medium was replaced by an equal volume of IS applied on ganglia for further 5 minutes and also collected. Levels of CGRP and PGE_2_ released by TG in medium were assessed by EIA.

### 3.1. Effect of NSAIDs on CGRP Release from Trigeminal Ganglia

**Figure 1:**
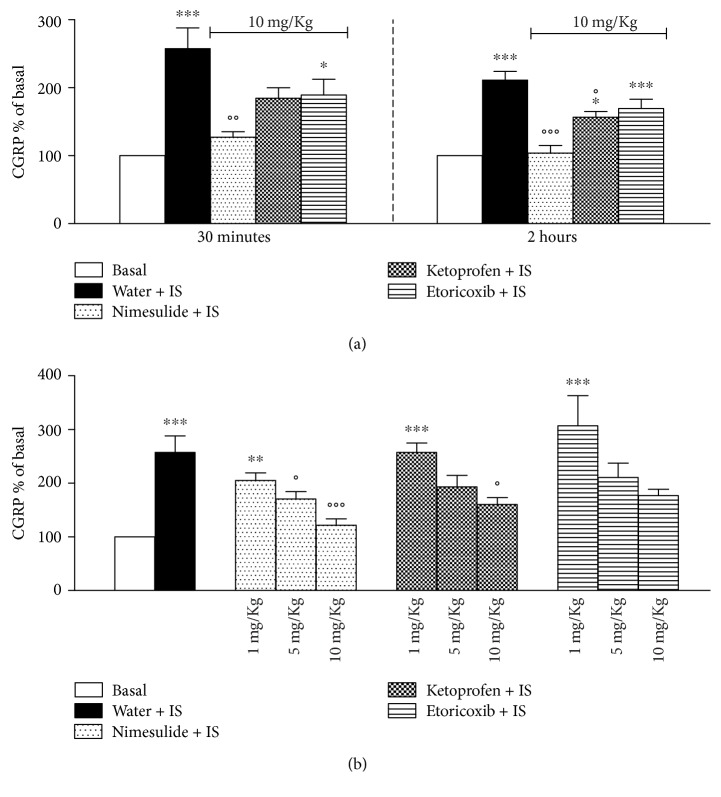
(a) Effect of oral administration of 10 mg/Kg nimesulide, ketoprofen, etoricoxib, or water on CGRP release from trigeminal ganglia after in vitro stimulation with inflammatory soup (IS). Thirty minutes and 2 hours after administration, rats were sacrificed, trigeminal ganglia were collected and maintained in vitro with DMEM alone for 5 minutes, then medium was collected, and ganglia were stimulated with IS for another 5 minutes. CGRP released in media basally and upon stimulation was measured by EIA. CGRP levels are expressed as % of basal values (measured before addition of IS) that were normalised to 100. Values are mean ± SEM of 6 animals. ^∗^*p* < 0,05; ^∗∗∗^*p* < 0,001 versus basal; °*p* < 0,05; °°*p* < 0,01; °°°*p* < 0,001 versus water + IS. (b) Dose-dependent inhibition of CGRP release in vitro by trigeminal ganglia after stimulation with IS by nimesulide, ketoprofen, and etoricoxib. Rats were treated orally with NSAIDs at the doses of 10, 5, and 1 mg/Kg. Two hours after administration, rats were sacrificed, and experiment was carried on as in (a). Values are mean ± SEM of 6 animals. ^∗∗^*p* < 0,01; ^∗∗∗^*p* < 0,001 versus basal; °*p* < 0,05; °°°*p* < 0,001 versus water + IS.

Figure 1(a) reports the effect of oral administration of NSAIDs at the dose of 10 mg/Kg on CGRP release. During the 5 minutes allowed for basal release, none of the drugs used altered basal CGRP level detected in media, which was 191 ± 12 pg/ml medium (mean ± SEM) for water-, 218 ± 21 pg/ml for nimesulide-, 162 ± 21.3 pg/ml for ketoprofen-, and 186 ± 13 pg/ml for etoricoxib-treated rats. In the figure, CGRP levels are expressed as % of basal values (measured before IS) that were normalised to 100. In vitro addition of IS significantly increased CGRP released from trigeminal ganglia. Thirty minutes after oral administration, only nimesulide was able to significantly decrease the IS-stimulated levels of CGRP in comparison with controls (water + IS). A partial inhibitory effect induced by ketoprofen was also observed, since CGRP concentrations were not anymore significantly different from basal levels, although no difference was present in respect to CGRP levels measured in water-treated animals after stimulation with IS. Two hours after in vivo drug administration, nimesulide completely blocked the IS stimulation of CGRP. At this time point also, ketoprofen was able to significantly reduce, although not completely, the IS-induced CGRP release. In contrast, no effect at any time point was observed in etoricoxib-treated rats.  Figure 1(b) reports the effect of escalating doses of the NSAIDs on IS-stimulated release, evaluated 2 hours after oral drug administration. Nimesulide was able to reduce CGRP-stimulated release also at 5 mg/Kg, but at the lowest dose of 1 mg/Kg, no significant reduction was observed. The effect of ketoprofen was significant only at the dose of 10 mg/Kg, while, as expected, etoricoxib did not modulate CGRP release.

### 3.2. Effect of NSAIDs on PGE_2_ Release from Trigeminal Ganglia

**Figure 2:**
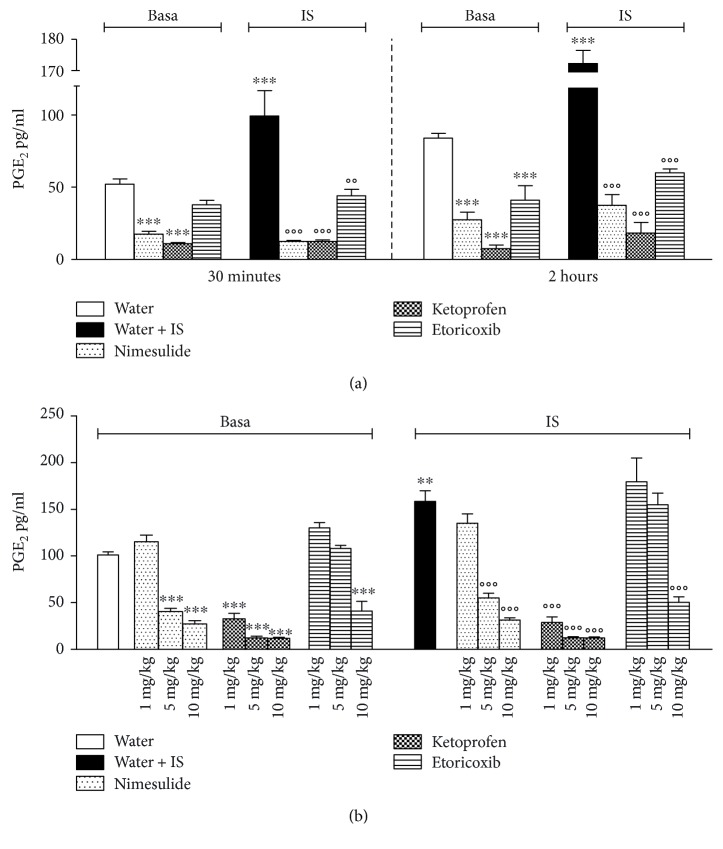
(a) Effect of oral administration of 10 mg/Kg nimesulide, ketoprofen, etoricoxib, or water on PGE_2_ release by trigeminal ganglia both in basal condition and after in vitro stimulation with inflammatory soup (IS). Thirty minutes and 2 hours after administration, rats were sacrificed, trigeminal ganglia were collected and maintained in vitro with DMEM alone for 5 minutes, then medium was collected, and ganglia were stimulated with inflammatory soup for another 5 minutes. Values are mean ± SEM of 6 animals; ^∗∗∗^*p* < 0,001 versus water; °°*p* < 0,01; °°°*p* < 0,001 versus water + IS. (b) Dose-response curve of the inhibition by nimesulide, ketoprofen, and etoricoxib treatments on in vitro basal and stimulated PGE_2_ releases by trigeminal ganglia. Rats were treated orally with NSAIDs at the doses of 10, 5, and 1 mg/Kg. Two hours after administration, rats were sacrificed, trigeminal ganglia were collected and treated as in (a). PGE_2_ release was measured in medium by EIA method. Values are mean ± SEM of 6 animals ^∗∗^*p* < 0,01, ^∗∗∗^*p* < 0,001 versus water; °°*p* < 0,01; °°°*p* < 0,001 versus water + IS.

Figure 2(a) shows basal- and IS-stimulated releases of PGE_2_ from TG obtained 30 minutes and 2 hours after administration of drugs at the dose of 10 mg/Kg. Thirty minutes after administration, nimesulide and ketoprofen significantly reduced PGE_2_ basal release, while etoricoxib was not effective. After IS stimulation, larger levels of PGE_2_ were released from TG, and all the drugs tested were able to significantly reduce them. Two hours after drug administration, TG isolated from rats treated with nimesulide, ketoprofen, and etoricoxib released lower amounts of PGE_2_, both in basal- and IS-stimulated conditions, in comparison with water-treated animals. With regard to the dose-response measured at 2 hours after treatment, ketoprofen significantly reduced both basal and stimulated PGE_2_ releases at all doses tested (1, 5, and 10 mg/Kg). The reduction induced by nimesulide was significant at 5 and 10 mg/Kg both for basal- and IS-activated releases, while etoricoxib significantly diminished PGE_2_ only at 10 mg/Kg (Figure 2(b)).

## 4. Discussion

In this work, we demonstrate for the first time that in vivo administration of nimesulide and ketoprofen inhibits stimulated in vitro CGRP release from dissected rat TG. The data described in this paper will contribute to the understanding of the pharmacological mechanisms of the perhaps most frequently used family of drugs in one of the most frequent painful conditions in which they are employed. NSAIDs in fact, despite the increasing use of the more recently introduced triptans, remain the most commonly used for migraine treatment, in particular for acute attacks, and they offer the most cost-effective therapy showing only minor side effects [17].

CGRP is known to play a key role in the pathophysiology of migraine and other types of headaches by being released at 3 different sites by TG neurons: the afferent terminals that innervate the meninges, the cell bodies in the ganglia, and the afferent fibers that synapse in the spinal cord and corelease glutamate, CGRP, and other peptides. In our study, we do not use cultured trigeminal cells, but dissected ganglia that are only divided in 4 pieces to aid diffusion. This approach allows the study of CGRP release from a TG where all the indirect and direct interactions of neurons with satellite cells are fundamentally maintained. Moreover, with this approach, the TG remain in vitro for a maximum of 15 minutes, avoiding prolonged cell culturing conditions that may affect the production and release of CGRP. It has in fact been demonstrated that cell culturing conditions can influence the expression and activity of CGRP in TG neurons [18–20]. Other studies have recently confirmed the advantage of using intact tissues, such as brainstem explants, rather than isolated cells for testing the effects of drugs on CGRP release in headache-related studies [21].

In this study, CGRP and PGE_2_ releases were tested in stimulated as well as basal conditions. Stimulation was used to reproduce the sensitization of the trigeminal system which is present in headache and migraine [22]. The IS employed to mimic trigeminal sensitization consisted of bradykinin, histamine, and serotonin, which are found to be released endogenously during inflammation, and elevated potassium and capsaicin were added to increase electrical activity, during neurogenic inflammation [23–25]. In the present study, NSAIDs are administered in vivo per os, in agreement with the route of administration used for these drugs by patients. The in vivo doses of nimesulide, ketoprofen, and etoricoxib that we chose have been validated in several models of inflammation and other pathological states in the rat, and we believe that we are reproducing in our experimental model the efficacious tissue level concentrations that are reached in human subjects [14, 15, 26–32]. We demonstrate that nimesulide and ketoprofen are able to reduce the stimulated release of CGRP from TG. We can therefore hypothesize that after treatment with these NSAIDs, the amount of CGRP available in the trigeminal vascular system both centrally and peripherally is reduced, allowing less sensitization and neurogenic inflammatory response. At the highest dose (10 mg/Kg), the inhibition induced by nimesulide is complete, indicating that the drug can indeed prevent the CGRP release. This effect is highly significant already at 30 minutes after oral administration, in line with the reported evidence of the fast onset of action of nimesulide in patients [16]. Interestingly, effective concentrations of nimesulide can be detected both in plasma and synovial fluids 30 min after drug intake in patients with osteoarthritis [14]. Although NSAIDs have been used clinically for many years, and it is well known that they share mechanisms that involve inhibition of different cyclooxygenases, it is not clear what other mechanisms are responsible for their different clinical profiles, with special reference to their use in the treatment of migraine. Experiments in rats or other animals as well as in human biopsies suggest that PGE_2_ released from rat dura mater upon electrical or chemical stimulations in vitro may play an important role in the pathogenesis of migraine [17, 33]. As expected, all the NSAIDs that we tested were able to rapidly inhibit both basal- and IS-stimulated PGE_2_ releases; however, some differences emerged, as the nonselective COX inhibitor ketoprofen reduced PGE_2_ release at the very low dose of 1 mg/Kg, the COX-2-selective etoricoxib was active only at the higher dose of 10 mg/Kg and the preferential COX-2 inhibitor nimesulide significantly reduced the PGE_2_ release at the doses of 5 and 10 mg/Kg. These observations suggest that both COX isoforms may be relevant in PGE_2_ production in the TG. The modulation by COX inhibitors of CGRP release from TG has not been studied previously in sufficient depth; although in recent studies, it was reported that COXs, and especially COX-2, may participate in CGRP release from TG induced by inflammatory stimuli, in different experimental models [13, 34–37]. Neeb et al. [13] demonstrated that the COX-2 inhibitor parecoxib was able to inhibit CGRP release from cultured trigeminal ganglia cells, while in our study, the selective COX-2 inhibitor etoricoxib was not effective. Several issues may explain this difference. Neeb et al. [13] added the NSAIDS directly on trigeminal ganglia cultures, while we preferred to administer them in vivo, in order to more closely mimic the clinical condition. Moreover, a relevant difference is the duration of stimulation. In our experiments, the IS was added only for 5 minutes, while Neeb et al. [13] demonstrated that a longer time is needed in order to activate COX-2 synthesis. It can therefore be hypothesized that mainly COX-1 may be involved in the early phases of TG stimulation, while COX-2 relevance is delayed. However, further experiments are needed in order to assess this hypothesis. Our present results indicate that the effect of NSAIDs on CGRP release in TG may not be related only to their ability to decrease PGE_2_. In fact, ketoprofen did not reduce CGRP release at doses of 1 and 5 mg/Kg, while at the same doses, it was able to inhibit PGE_2_ release, while etoricoxib at 10 mg/Kg inhibited PGE_2_ release, but did not influence CGRP release. Of the 3 drugs studied, nimesulide was the only NSAID able to reduce CGRP at the dose of 5 mg/Kg; at this dose, nimesulide induced a PGE_2_ inhibition that was similar to that of ketoprofen at the lowest dose at which no effect on CGRP was observed. Moreover, while all drugs reduced PGE_2_ at the earliest time tested (30 minutes after oral administration), only nimesulide was able to modulate CGRP at this point in time. Based on these evidences, it can be speculated that also other different mechanisms, besides PGE_2_ inhibition, may be involved in the effects of NSAIDs on CGRP release. This kind of dissociation is particularly evident for nimesulide. These results are in agreement with our previous data indicating that nimesulide is more active than other NSAIDs in the inhibition of stimulated substance P release from rat sensory neurons and further confirm the multifactorial mode of action of this NSAID [38–41]. The signals and mechanisms governing the release of CGRP from sensory neurons are not fully elucidated. Recently, it has been suggested that CGRP release from TG takes place via two independent mechanisms: a calcium/Snap 25-dependent as well as a calcium-independent mechanism involving increases in intracellular sodium ions and activation of the acid sensitive ion channel ASIC3 [36]. It is interesting to note that many of the proinflammatory mediators that promote and maintain neurogenic inflammation within the dura mater have been shown to stimulate expression and activity of these channels and that NSAIDs at therapeutic doses inhibit ASIC channel activity [42, 43]. More specific studies are necessary to identify the molecular mechanisms of the effects of nimesulide and ketoprofen on CGRP release from TG.

## 5. Conclusions

The evaluation of basal and stimulated in vitro CGRP release from rodent-isolated trigeminal ganglia is a reliable method for the study of the effects of drugs used in the treatment of migraine. The efficacy in reducing CGRP release contributes a rationale for choosing a NSAID in the treatment of migraine. The rapid onset of the effect of nimesulide may explain the large use of this NSAID by patients with migraine [44].
